# RGS10 controls the metabolic and neuroinflammatory benefits of NLRP3 suppression in diet-induced obesity in male mice

**DOI:** 10.21203/rs.3.rs-10554888/v1

**Published:** 2026-09-02

**Authors:** Declan J. Gresham, Jaegwon Chung, Jung Ha Byun, Yu Zhang, Nicole Zhang, Yoon-Seong Kim, Adetutu Adebowale, Jina Lim, Kelly Menees, Nhi Diep, Matthew C. Havrda, Jae-Kyung Lee

**Affiliations:** 1 Department of Physiology and Pharmacology, University of Georgia College of Veterinary Medicine, Athens, GA, USA.; 2 Intergrative Physiology and Pharmacology program, University of Georgia, Athens, GA, USA.; 3 Neuroscience program, University of Georgia, Athens, GA, USA; 4 Robert Wood Johnson Medical School Institute for Neurological Therapeutics, Department of Neurology, Rutgers Biomedical and Health Sciences, Piscataway, New Jersey 08854, USA; 5 Department of Biomedical Sciences, University of New England College of Osteopathic Medicine, Biddeford, ME, USA

## Abstract

The NLRP3 inflammasome is a central mediator of metabolic inflammation, yet the upstream mechanisms remain poorly defined. We identified RGS10 as a critical regulator of NLRP3-driven immunometabolic responses. In macrophages, loss of RGS10 enhanced calcium-dependent NLRP3 inflammasome activity during both priming and activation. In high-fat diet-fed male mice, *Nlrp3* deletion improved glucose tolerance, adipose remodeling, and hepatic and hypothalamic pathology, whereas *Rgs10* deletion induced the opposite phenotype. These benefits required RGS10: in *Nlrp3*^*−/−*^/*Rgs10*^*−/−*^ (dKO) males, the improvements in glucose tolerance, adipose remodeling, and hypothalamic microgliosis were abolished. This requirement was selective; RGS10 acted dominantly to restrain myeloid and hepatic inflammation independent of NLRP3, and the interaction was sex-dependent. Finally, RGS10 declined in aged and Parkinson’s disease human microglia, and aged *Rgs10*^*−/−*^ mice developed glucose intolerance. Together, these findings establish RGS10 as a dominant, sex-dependent upstream regulator of NLPR3-driven inflammation, suggesting that age- or inflammation-associated RGS10 loss may limit the efficacy of NLRP3-targeted therapies.

## INTRODUCTION

Chronic low-grade inflammation has been recognized as the common driver of many diseases, linking immune dysregulation and metabolic dysfunction to the pathogenesis of many disorders, including obesity, type 2 diabetes (T2DM), nonalcoholic fatty liver disease (NAFLD), cardiovascular disease (CVD), and neurodegenerative disorders^1–4^. The nucleotide-binding domain, leucine-rich-containing, pyrin domain-containing-3 (NLRP3) inflammasome is a multimeric protein complex part of the innate immune system that serves as an intracellular sensor of various pathogen-associated molecular patterns (PAMPs) and damage-associated molecular patterns (DAMPs). NLRP3 is highly expressed in immune cells, particularly in monocytes and macrophages^1^. Inflammasome activation results in cleavage and release of IL-1β and IL-18 and pyroptotic cell death in response to various cellular stressors, including mitochondrial reactive oxygen species (ROS) and saturated fatty acids, which are elevated in obesity^2,3^. Several preclinical studies have shown that loss of NLRP3 protects against high-fat diet (HFD)-induced glucose intolerance, insulin resistance, and adiposity^3,4^, while NLRP3 inhibition in mice reduced HFD-induced weight gain, systemic inflammation, steatosis, and hypothalamic astrogliosis^5–7^.

Regulator of G protein signaling (RGS) proteins are a family of GTPase-activating proteins (GAPs) that negatively regulate G protein-coupled receptor (GPCR) signaling by binding to specific Gα subunits^8^. Several RGS proteins have been implicated in both positive and negative regulation of HFD-induced inflammation, obesity, NAFLD, and insulin resistance. Studies have demonstrated that RGS5 protects against liver steatosis and fibrosis^9–11^, while RGS4 and RGS16 maintain fatty acid and glucose homeostasis and support insulin secretion^12,13^. Other RGS proteins, such as RGS6 and RGS2, exacerbate NAFLD and insulin resistance^14,15^. Thus, there is growing evidence that RGS proteins regulate metabolic dysfunction and systemic inflammation, making them potential therapeutic targets for treating obesity, insulin resistance, and NAFLD.

RGS10 belongs to the R12 subfamily and is one of the smallest members of the RGS family; it binds to Gα_i_, Gα_z_, and Gα_q_^16^. High levels of RGS10 expression have been detected in the brain, bone marrow, and lymph nodes; immune cells, particularly myeloid cells, are the main cell types with enriched RGS10 expression^1,17^. RGS10 expression is dynamically regulated during inflammatory responses. Lipopolysaccharide (LPS) stimulation promotes histone deacetylation-mediated epigenetic repression of *Rgs10* transcription in myeloid cells^18,19^. In addition, aging alters RGS10 expression in immune populations, coincident with age-dependent shifts in peripheral immune subsets^20^. We have previously reported that *Rgs10*^−/−^ mice fed with HFD exhibit increased weight gain, glucose intolerance, insulin resistance, and systemic inflammation^21^. Furthermore, both RGS10 and NLRP3 have recently been identified as potential therapeutic targets for T2DM^22^. Given the central role of NLRP3 in the pathogenesis of metabolic disease, RGS10 may act as an endogenous brake on chronic metabolic inflammation. However, whether RGS10 regulates NLRP3 inflammasome signaling and contributes to the metabolic benefits associated with NLRP3 suppression remains unknown. Here, we investigated the role of RGS10 in regulating NLRP3 inflammasome activity and metabolic inflammation. Using bone marrow-derived macrophages and genetic models of NLRP3 and RGS10 deficiency, we demonstrate that RGS10 negatively regulates both the priming and activation phases of the NLRP3 inflammasome. In HFD-fed mice, NLRP3 deficiency improved metabolic and inflammatory outcomes in the liver, adipose tissues, and brain; however, these protective effects were largely abolished in the absence of RGS10. Together, these findings reveal a previously unrecognized functional interaction between RGS10 and the NLRP3 inflammasome that regulates metabolic inflammation in diet-induced obesity.

## Results

### RGS10 deficiency enhances NLRP3 inflammasome priming and activation in BMDMs in a calcium-dependent manner.

Publicly available human single-cell RNA sequencing (scRNAseq) data from the Broad Institute Single Cell Portal shows that RGS10 is highly expressed in immune cells, particularly in myeloid cells, with high co-expression with NLRP3 inflammasome components in monocyte and macrophage populations^23,24^ (Figure 1A). Building on our prior findings that RGS10 suppresses NF-κB-dependent proinflammatory activation in myeloid cells^17,25–27^, we examined whether RGS10 also regulates NLRP3 inflammasome priming in macrophages. Bone marrow-derived macrophages (BMDMs) were generated from wild-type (WT) and *Rgs10*^*−/−*^ mice and validated for purity by flow cytometry **(Figure S1)** and stimulated with LPS. As expected, LPS induced NLRP3 and pro-IL-1β expression in both genotypes and RGS10 deficiency further augmented this priming, with *Rgs10*^*−/−*^ BMDMs significantly higher pro-IL-1β (2.27-fold, *p*<0.0001) than WT (Figure 1B–D). Pro-gasdermin-D (GSDMD), which is constitutively expressed, exhibited a higher baseline level in *Rgs10*^*−/−*^ BMDMs that remained unchanged following LPS priming (Figure 1B and E). Thus, loss of RGS10 potentiates NLRP3 inflammasome priming.

We next determined whether elevated priming in *Rgs10*^*−/−*^ BMDMs translated into elevated inflammasome activation. BMDMs were primed with LPS (100 ng/mL, 3 h) and stimulated with ATP (5 mM, 2hrs) or nigericin (NG, 5uM, 1 h), and lysates and supernatants were analyzed (Figure 1F). *Rgs10*^*−/−*^ BMDMs secreted significantly higher cleaved IL-1β (p17) in response to both ATP and NG, with correspondingly higher cleaved caspase-1 (p20) (Figure 1H). ELISA analyses of confirmed significantly increased IL-1β secretion from *Rgs10*^−/−^ BMDMs after both stimuli, up to 2.22-fold (*p*<0.0001) (Figure 1I). Thus, RGS10 negatively regulate both the priming and activation phases of the NLRP3 inflammasome in male macrophages.

Because RGS10 regulates store-operated calcium entry^28^, which influences NF-κB activity^29^ and induces NLRP3 activation^30–32^, we tested whether calcium is required for RGS10-mediated inhibition. In calcium-free medium, the increased priming caused by RGS10 deficiency in male BMDMs was lost and reversed. *Rgs10*^*−/−*^ BMDMs expressed significantly lower NLRP3 than WT after LPS, with no difference in pro-IL-1β (Figure 1J–M). Thus, calcium is required for RGS10-mediated inhibition of inflammasome priming.

We next tested whether extracellular calcium is required for RGS10 to restrain inflammasome activation, treating BMDMs with LPS and ATP in normal or calcium-free medium (Figure 1N). In male supernatants, RGS10 loss significantly increased IL-1β p17 (2.00-fold, *p*=0.0044) (Figure 1O and P). Caspase-1 p33, the fully active transient species^33^, was significantly higher in *Rgs10*^*−/−*^ supernatants and abolished without calcium; cleaved caspase-1 (p20) showed the same pattern (Figure 1Q–S). Thus, calcium signaling is required for the increased IL-1β cleavage and caspase-1 activation in male *Rgs10*^*−/−*^ BMDMs, indicating that RGS10 suppresses inflammasome activity by modulating calcium flux.

LPS-induced priming and ATP-induced activation were also assessed in female BMDMs **(Figure S2)**. RGS10 loss increased pro-GSDMD during priming in a calcium-dependent manner (2.15-fold, p=0.0058) but did not significantly alter NLRP3 or pro-IL-1β and did not potentiate the activation phase **(Figure S2)**. Thus, the calcium-dependent mechanism by which RGS10 restrains NLRP3 is conserved across sexes, whereas the magnitude of the inflammasome response, particularly at the activation phase, is male-predominant.

#### RGS10 is required for the metabolic benefits of NLRP3 deficiency during HFD in a sex-specific manner

Previously, *Rgs10*^*−/−*^ mice exhibited exacerbated diet-induced weight gain, glucose intolerance, and insulin insensitivity^21^, whereas independent studies have shown protection in that *Nlrp3*^*−/−*^ mice are protected from HFD-induced metabolic dysfunction^4^. To test whether RGS10’s negative regulation of NLRP3 influences susceptibility to HFD-induced obesity and metabolic dysfunction, we generated *Nlrp3*^*−/−*^/*Rgs10*^*−/−*^ double-knockout (dKO) mice by crossing *Nlrp3*^*−/−*^ and *Rgs10*^*−/−*^ strains (Figure 2A) and validated genotypes by PCR (Figure 2B). WT, *Nlrp3*^*−/−*^, *Rgs10*^*−/−*^, and dKO mice were fed HFD for 12 weeks, and weight gain, food intake, and final change in body weight were monitored (Figure 2C–E, **S3A-C)**. In males, *Rgs10*^*−/−*^ mice again displayed significantly increased HFD-induced weight gain, gaining 35.1% more weight than WT *(p=*0.0006) (Figure 2C, E). Conversely, *Nlrp3*^−/−^ mice were protected, with reduced final weight. dKO males exhibited an intermediate phenotype: weight gain was comparable to WT yet remained significantly higher than *Nlrp3*^−/−^ mice, indicating that the reduced weight gain observed in *Nlrp3*^−/−^ mice is partially dependent on RGS10. Food consumption did not differ significantly among male groups (Figure 2D). We next performed glucose tolerance tests (GTT) and insulin sensitivity tests (IST) in male WT, *Nlrp3*^*−/−*^*, Rgs10*^*−/−*^, and dKO mice. Under normal chow (NC), *Rgs10*^*−/−*^ mice displayed significantly increased glucose area under the curve (AUC) relative to WT, indicating impaired glucose homeostasis independent of diet (Figure 2F, G). Under HFD, Nlrp3^−/−^ mice showed the lowest glucose AUC of all groups; this critical benefit was abolished in dKO, which showed significantly higher AUC than *Nlrp3*^*−/−*^ mice (*p*=0.0143) and reverted to WT level (Figure 2H, I). Thus, the glucose-tolerance benefit of NLRP3 deficiency requires RGS10. Insulin sensitivity followed a related pattern. Under NC, *Rgs10*^*−/−*^ mice showed impaired insulin responsiveness, with significantly increased IST AUC vs WT (Figure 2J, K). Under HFD, *Nlrp3*^−/−^ mice showed improved early glucose disposal, though overall IST AUC did not differ among groups (Figure 2L, M). Thus, in male mice, RGS10 is required to maintain glucose tolerance and insulin sensitivity independently of diet, while the glycemic benefit of NLRP3 is RGS10-dependent, fully for glucose tolerance and partially for weight gain.

In females, the RGS10-NLRP3 interaction diverged from that of males **(Figure S3).** RGS10 loss again exacerbated HFD-induced weight gain in both *Rgs10*^*−/−*^ (61.4%, *p*=0.0333) and dKO (56.9%, *p*=0.1102), despite reduced food intake, whereas *Nlrp3*^*−/−*^ deletion did not protect females from HFD-induced weight gain, unlike males. NLRP3 deficiency improved female glucose tolerance (AUC reduced vs WT, *p*=0.0317) and insulin sensitivity, but combined deficiency did not simply abolish these benefits: dKO females developed glucose intolerance exceeding all other genotypes **(Figure S3G)**, while insulin sensitivity returned to WT levels **(Figure S3K)**. The glucose intolerance and insulin resistance seen in *Rgs10*^*−/−*^ males were absent in females. Thus, the metabolic RGS10-NLRP3 interaction is sex-dependent, with epistatic loss of protection in males vs synergistic dysfunction in females. Because the phenotypes are more robust and interpretable in males, subsequent experiments focused on male cohorts.

### NLRP3 deficiency protects against HFD-induced hepatic steatosis in an RGS10-dependent manner.

To determine whether RGS10 influences hepatic steatosis via the regulation of the NLRP3 inflammasome, we performed histological, quantitative real-time PCR (RT-qPCR), and Western blot analyses of livers from male WT, *Nlrp3*^*−/−*^, *Rgs10*^*−/−*^, and dKO mice after 12 weeks of NC or HFD, quantifying total vacuolar area, macrovesicular vacuolar (MVA), and non-macrovesicular vacuolar area (NMVA) by pixel classification (Figure 3B, C). Even under NC, *Rgs10*^*−/−*^ and dKO mice showed increased liver weight (*p*=0.0007 and *p*=0.0002 vs WT) and increased vacuolar area relative to WT **(Figure S4)**, indicating that RGS10 loss promotes hepatic pathology independently of diet. Under HFD diet, *Rgs10*^*−/−*^ males displayed markedly increased liver weight (37.9% over WT, *p*=0.0194) and the highest total vacuolar area and NMVA of all groups, whereas *Nlrp3*^*−/−*^ mice showed protection, with significantly reduced total vacuolar area, MVA, and NMVA (Figure 3A, D). Importantly, these protective effects of *Nlrp3* deletion were only partially retained in dKO mice. In dKO livers, all three measures were similar to WT, yet all were significantly higher than *Nlrp3*^*−/−*^ (*p*=0.0002, *p*=0.0008, and *p*<0.0001, respectively). Thus, the hepatoprotective effects of NLRP3 deficiency against HFD-induced steatosis require RGS10.

Consistent with reduced lipid accumulation, *Fasn*, a lipogenic enzyme whose expression correlates with hepatic steatosis but not inflammation^34^, was significantly downregulated in *Nlrp3*^−/−^ and dKO mice (Figure 3E). *Rgs10*^−/−^ mice show only trends towards increased profibrotic gene expression, whereas *Nlrp3*^*−/−*^ mice displayed robust anti-fibrotic signatures, with significantly reduced *Tgfb1, Col1a1, Col4a1*, and *Acta2* (Figure 3F–I). These reductions were not simply lost but reversed in dKO mice, in which both *Tgfb1* and *Col4a1* were significantly upregulated above WT (*p*=0.021 and *p*=0.0007, respectively), indicating that combined loss of RGS10 and NLRP3 synergistically derepresses fibrotic gene expression rather than reflecting simple loss of an RGS10-dependent benefit.

Hepatic inflammatory and myeloid activation markers were assessed by RT-qPCR. *Cd68*, a marker of macrophage abundance, was significantly decreased in *Nlrp3*^*−/−*^ mice but elevated above WT in dKO mice (Figure 4A). *Cd11b* expression was increased in both *Rgs10*^−/−^ (2.1-fold, *p*<0.0001) and dKO mice (1.75-fold, *p*=0.0011) (Figure 4B). Similarly, *Nlrp3*^−/−^ mice showed reduced *Ccl2*, whereas dKO mice showed elevated *Ccl2*, and *Tnf* was elevated in both *Rgs10*^−/−^ and dKO mice, reaching 2.00-fold in dKO (*p*<0.0001) (Figure 4C, D). Thus, as with the fibrotic genes, hepatic inflammatory gene expression in dKO mice was driven above WT levels rather than returning to it, consistent with RGS10 restraining hepatic inflammation largely independently of NLRP3. Hepatic inflammation was further analyzed by Western blot (Figure 4E). As expected, NLRP3 protein was detected only in WT and *Rgs10*^*−/−*^ mice, with no significant differences in expression between these two groups (Figure 4E, F). NF-κB p65 phosphorylation was significantly elevated only in *Rgs10*^*−/−*^ livers relative to WT mice, while IκBa levels did not differ among the 4 genotypes (Figure 4G, H). At the protein level, CD68 was significantly increased in *Rgs10*^−/−^ mice, with a nonsignificant trend toward increased expression in dKO mice (Figure 4I). Because HFD impairs hepatic autophagy and increase p62 phosphorylation^35,36^, we assessed phospho-p62/p62 ratios: these were reduced in *Nlrp3*^−/−^ mice relative to *Rgs10*^*−/−*^ mice (2.95-fold, *p*=0.05), consistent with improved autophagic function, whereas dKO mice showed a trend toward increased phospho-p62/p62 relative to *Nlrp3*^−/−^ mice (Figure 4J). Given that these differences were borderline and based on a single autophagy marker, this observation should be interpreted with caution. Collectively, NLRP3 deficiency protected against HFD-induced liver steatosis, fibrosis, inflammatory gene expression, and, tentatively, autophagy impairment, but the dependence on RGS10 differed by readout. The steatosis benefit was partially lost in dKO livers (intermediate between *Nlrp3*^−/−^ and WT), indicating RGS10dependence, whereas profibrotic and proinflammatory gene expression in dKO mice was driven to or above WT levels, indicating synergistic derepression rather than simple loss of an RGS10-dependent benefit. Thus, RGS10 is specifically required for the steatosis-protective effect of NLRP3 deficiency, while dominantly restraining hepatic fibrotic and inflammatory responses, consistent with the systemic and tissue-level patterns observed in Figure 2 and 5.

#### RGS10 shapes adipose myeloid composition independently of NLRP3 and restrains adipose inflammation during HFD.

To assess HFD-induced adipose tissue hypertrophy, we performed histological analyses of white adipose tissues from mice after 12 weeks of HFD feeding. On average, inguinal white adipose tissue (ingWAT) mass was increased by 47.6% in *Rgs10*^*−/−*^ mice (*p*=0.0008) vs WT (Figure 5A). Despite RGS10 deficiency, dKO mice showed significant reductions in both ingWAT and epididymal WAT (eWAT) mass compared to *Rgs10*^*−/−*^ mice (Figure 5B). By H&E *Rgs10*^*−/−*^ mice displayed markedly enlarged adipocytes with significantly increased adipocyte area, consistent with exacerbated adipocyte hypertrophy (Figure 5C, D). In contrast, *Nlrp3*^*−/−*^ mice exhibited reduced adipocyte area and increased adipocyte number, indicating reduced adipocyte hypertrophy and improved remodeling (Figure 5E). In dKO mice, adipocyte area returned to WT levels while adipocyte number was significantly higher than *Rgs10*^*−/−*^ mice, indicating partial reductions in adipose hypertrophy.

To probe underlying mechanisms, metabolic and thermogenic genes were measured in ingWAT by RT-qPCR. Because the NLRP3 inflammasome suppresses PPARg expression and adipogenesis^37^, we examined these programs. Consistent with prior work, *Nlrp3*^*−/−*^ mice showed increased *Pparg*, whereas *Pparg* and *Adipoq* (adiponectin) were significantly reduced in both *Rgs10*^−/−^ and dKO mice (Figure 5F, G). *Glut4* was highest in *Nlrp3*^*−/−*^ mice and significantly downregulated in dKO mice (Figure 5H). *Prdm16* followed a similar pattern, with dKO mice significantly reduced below all other groups (Figure 5I). Most strikingly, *Ucp1* was dramatically upregulated in *Nlrp3*^*−/−*^ mice (3.39-fold, *p*<0.0001), an induction completely abolished in dKO mice (Figure 5J). Thus, NLRP3 deficiency improves adipose tissue remodeling during HFD, reducing adipocyte hypertrophy and enhancing adipogenic and thermogenic gene programs, and these benefits are lost in dKO mice, indicating that the metabolic remodeling benefits of *Nlrp3* deletion are RGS10-dependent.

Adipose Inflammatory phenotypes were assessed by RT-qPCR and Western blot. *Rgs10*^−/−^ mice showed significantly elevated *Cd68* and *Cd11b*, whereas *Nlrp3*^*−/−*^ mice showed reduced *Cd68*; in dKO mice, *Cd68* mRNA was modestly but significantly elevated above WT (Figure 5K, L). At the protein level, *Rgs10*^*−/−*^ mice showed a trend toward increased CD68 and significantly elevated phospho-p65/p65, and dKO mice showed the highest CD68 protein of all groups (4.37-fold vs WT, *p*=0.0095) (Figure 5M–O). Thus, RGS10 restrains adipose inflammation largely independently of NLRP3: loss of RGS10 increased inflammatory gene expression and NF-κB activation, and the elevated macrophage markers persisted in dKO mice, whereas NLRP3 deficiency alone did not reduce them. In contrast to the RGS10-dependent remodeling benefits, adipose inflammatory signaling is dominated by RGS10 and is not a downstream consequence of NLRP3 activity. Notably, dKO mice exhibited elevated CD68 protein despite WT-level NF-κB p65 phosphorylation, suggesting accumulated macrophage infiltration independent of steady-state NF-κB signaling at this point.

#### RGS10 drives adipose myeloid polarization independently of NLRP3 but is required for the anti-inflammatory monocyte effect of NLRP3 deficiency.

To assess how loss of RGS10 and NLRP3 influences HFD-induced adipose tissue myeloid cell phenotypes and infiltration, we performed flow cytometry analyses on stromal vascular fractions (SVF) from ingWAT and eWAT of male mice after 12 weeks of HFD (gating in Figure 6A). Ly6G^+^ neutrophils, which have been implicated in obesity-associated adipose tissue inflammation^38^, were increased in dKO, while total CD11b^+^/CD115^+^ monocyte/macrophage frequency was modestly reduced (13.2%, *p*=0.0062), suggesting reduced recruitment into adipose tissue **(Figure S5)**.

Monocyte/macrophage polarization was characterized by expression of CD11c and CD206 (Figure 6B). CD11c^+^/CD206^−^ cells were classified as proinflammatory, CD11c^−^/CD206^+^ as antiinflammatory, and CD11c^+^/CD206^+^ cells were defined as HFD-associated macrophages, a population previously shown to be proinflammatory^39^. Proinflammatory CD11c^+^/CD206^−^ frequencies were significantly increased in both *Rgs10*^*−/−*^ and dKO mice (Figure 6C), indicating that this polarization shift was driven by RGS10 loss independently of NLRP3. The CD11c^−^/CD206^−^ double-negative population showed the reciprocal pattern, highest in *Nlrp3*^*−/−*^ and lowest in *Rgs10*^−^*/*^−^ and dKO mice. No differences were observed in anti-inflammatory CD11c^−^/CD206^+^ macrophages. Knockout of NLRP3 reduced HFD-associated CD11c^+^/CD206^+^ macrophages, but this was lost in dKO mice. Monocyte activation was evaluated by Ly6C expression (Figure 6D). *Nlrp3*^*−/−*^ mice exhibited a 5.3% reduction in Ly6C^Hi^ monocytes (*p*=0.0101) vs WT, indicating reduced proinflammatory monocyte activation; this effect was abolished in dKO mice (Figure 6E), indicating that the reduction in proinflammatory monocytes conferred by NLRP3 deficiency required RGS10. Ly6C^Lo^ frequencies did not differ among groups.

### RGS10 is required for the suppression of hypothalamic gliosis conferred by NLRP3 deficiency in HFD-fed mice.

Because NLRP3 inhibition reduces hypothalamic gliosis^5,6^, which contributes to weight gain^40–43^, we asked whether RGS10 regulates NLRP3-mediated gliosis, characterizing activated microglia (Iba1) and astrocytes (GFAP) by immunofluorescence in the arcuate nucleus (ARC), dorsomedial hypothalamus (DMH), and ventromedial hypothalamus (VMH) (Figure 7A). In the ARC, *Rgs10* deletion significantly increased Iba1+ area vs WT while *Nlrp3* deletion significantly reduced it (Figure 7B, C). Notably, this reduction was abolished in dKO mice, which showed significantly higher Iba1+ area than *Nlrp3*^*−/−*^ mice (p=0.0030). Neither single deletion altered GFAP+ area in the ARC, but dKO mice displayed significantly elevated GFAP+ area vs. WT. In the DMH, NLRP3 deficiency significantly reduced both Iba1 and GFAP staining vs. WT (Figure 7D, E). dKO mice displayed significantly higher Iba1+ area than *Nlrp3*^*−/−*^ mice, but like *Nlrp3*^*−/−*^ mice, retained reduced GFAP+ area (p=0.0019) vs. WT. In the VMH, where baseline gliosis was low, neither single deletion altered microgliosis, but combined deficiency elevated Iba1^+^ area above WT, mirroring the synergistic pattern seen for astrocytes and peripheral inflammation; GFAP^+^ area did not differ among the four groups **(Figure S6)**. Together, NLRP3 deficiency reduced hypothalamic microgliosis in both the ARC and DMH, and this reduction was lost in dKO mice, indicating that RGS10 is required for the microglial anti-inflammatory effect of NLRP3 deficiency in these regions. In the VMH, by contrast, neither single deletion led to microgliosis, but combined deficiency elevated Iba1^+^ area above WT, indicating a synergistic rather than epistatic interaction. The astrocyte responses in the ARC were likewise elevated only with combined deficiency, while the NLRP3-associated reduction in DMH astrogliosis persisted in dKO mice, indicating that RGS10 regulates astrocytic responses in a region-specific manner.

#### RGS10 declines in human microglia with aging and disease, and its loss drives age-related glucose intolerance.

RGS10 is highly expressed in myeloid cells such as microglia and macrophages^1,17^. To assess the human relevance of RGS10 loss, we reanalyzed published single-nucleus RNA sequencing (snRNA-seq) data from human microglia in the midbrain and found that *RGS10* expression decreases along the combined pseudopathology (cPP) trajectory, which captures the transcriptomic transition from homeostatic microglia to aging- and PD-associated inflammatory states^44^ (Figure 8A). *RGS10* was among the most significantly downregulated genes, together with neurodegeneration-associated genes such as *BIN1* (Figure 8B). These data identify *RGS10* as a myeloid homeostatic gene that is lost with aging and inflammation in humans.

Because chronic CNS and systemic inflammation and metabolic dysfunction are implicated in neurodegeneration^45^, we asked whether age-related RGS10 loss produces a metabolic phenotype. Aged (17–21 months) *Rgs10*^*−/−*^ mice of both males and females gained significantly more weight than age-matched WT (Figure 8C and D), indicating an age-dependent phenotype. Following oral glucose challenge, aged *Rgs10*^*−/−*^ mice of both sexes showed significantly elevated blood glucose and trends of increased AUC relative to WT (males, Figure 8E, F; females, Figure 8G, H), demonstrating that glucose intolerance emerges with age in both sexes. To determine whether this glucose intolerance reflected impaired insulin sensitivity, we performed IST tests. Strikingly, the response was sex-dimorphic: aged female *Rgs10*^−*/*−^ mice developed pronounced insulin resistance, with significantly elevated glucose across multiple time points following insulin challenge (Figure 8K) and significantly higher AUC (Figure 8L), whereas aged male *Rgs10*^−*/*−^ mice retained normal insulin sensitivity, indistinguishable from WT (Figure 8I, J). Thus, aged *Rgs10*^−*/*−^ mice develop glucose intolerance in both sexes, but through distinct mechanisms: insulin resistance in females and an insulin-independent process in males, consistent with impaired insulin secretion. Together, these findings link RGS10 to human microglial aging and disease and establish that its loss drives age-related metabolic dysfunction through sex-dependent mechanisms.

## Discussion

Our previous work demonstrated that RGS10 attenuates proinflammatory activation in myeloid cells^17,25–27^ and protects against HFD-induced metabolic dysfunction^21^. Loss of RGS10 skews both myeloid and lymphoid populations toward proinflammatory phenotypes and exacerbates pathology across preclinical models, including colitis, rheumatoid arthritis, and influenza infection^46–49^. The current study extends these findings by identifying RGS10 as a negative regulator of NLRP3 inflammasome priming and activation in macrophages (Figure 1). Genetic deletion or pharmacologic inhibition of NLRP3 improves glucose homeostasis, reduces adiposity, attenuates inflammation in the brain and periphery, and protects adipose tissue and liver homeostasis in preclinical models of obesity and metabolic disease^3–7,37^. Consistent with these reports, our data support a model in which HFD-induced NLRP3 activation drives insulin resistance, steatosis, adipose tissue dysfunction, and hypothalamic gliosis.

Importantly, the metabolic benefits of NLRP3 deficiency depend on RGS10, and this dependence is sex-specific. In males, NLRP3 deficiency improved glucose tolerance, and this benefit was abolished in dKO mice, while the reduction in weight gain was only partially lost (Figure 2). In females, by contrast, dKO mice exhibited the most severe glucose intolerance of any genotype **(Figure S3)**, indicating that combined loss is synergistically detrimental rather than reflecting simple loss of an RGS10-dependent benefit. Additionally, Sex differences also extended to inflammasome priming and activation. RGS10 deficiency potentiated priming and activation broadly in male BMDMs, but only pro-GSDMD in females with no significant potentiation of the activation phase **(Figure S2)**. Sex hormones exert differential effects in diet-induced obesity and inflammation^50^ and estrogen has been shown to either enhance or suppress inflammasome activity depending on context^51^. One HFD study reported that comparable adiposity developed later in females, with males alone showing upregulated inflammasome activity in visceral adipose myeloid cells^52^, consistent with our findings that NLRP3-dependent metabolic phenotypes are robust in male mice. Experiments using aged or gonadectomized mice will be important to determine how aging and loss of sex hormones influence the metabolic effects of NLRP3 inhibition.

In the liver, NLRP3 and RGS10 regulated HFD-induced pathology through distinct relationships depending on the readout. Consistent with prior studies implicating the NLRP3 inflammasome in metabolic liver diseases^3,4,7,37^, genetic ablation of *Nlrp3* reduced liver mass and attenuated hepatic steatosis, fibrotic gene expression, and inflammatory phenotypes (Figures 3 and 4). This protection was RGS10-dependent: dKO mice lost the reduction in hepatic vacuolar area conferred by NLRP3 deficiency, returning toward WT levels (Figure 3D). The non-macrovesicular vacuolar compartment quantified here is consistent with microvesicular vacuole and hydropic changes and may in part reflect ballooning degeneration; formal assessment of hepatocyte ballooning would require pathologist-scored histology using established semiquantitative criteria. In contrast, hepatic inflammation followed a different pattern. Loss of RGS10 alone markedly elevated inflammatory gene expression. Rather than *Nlrp3* deletion protection being simply lost without RGS10, the combined deficiency was synergistic, indicating that RGS10 and NLRP3 independently restrain hepatic inflammation and that RGS10 is the dominant regulator (Figure 4A–D). A similar pattern was seen for fibrotic gene expression (Figure 3F and H). Together, these liver findings indicate that RGS10 exerts dominant control over HFD-induced hepatic inflammation and fibrotic signaling, functioning largely independent of NLRP3, while being specifically required for the steatosis-protective effect of NLRP3 deficiency.

In adipose tissue, NLRP3 deficiency promoted a metabolically favorable remodeling phenotype, characterized by reduced adipose hypertrophy, increased adipocyte number, and enhanced adipogenic and thermogenic gene programs (*Pparg*, *Ucp1*) (Figure 5). These remodeling benefits were RGS10-dependent, as they were lost in dKO mice, which resembled WT or *Rgs10*^−*/*−^ mice (Figure 5D–J). In contrast, adipose inflammation followed a distinct pattern: loss of RGS10 drove proinflammatory gene expression, NF-κB activation, and CD11c^+^/CD206^−^ myeloid polarization, with dKO mice largely phenocopying *Rgs10*^−*/*−^ mice independently of NLRP3 (Figures 5K–O, 6C). Notably, the reduction in proinflammatory Ly6C^hi^ monocytes conferred by NLRP3 deficiency was abolished in dKO mice (Figure 6E), indicating that this specific anti-inflammatory benefit required RGS10. Thus, in adipose tissue as in the liver, RGS10 was required for the metabolic remodeling benefits of NLRP3 deficiency while independently dominating myeloid inflammatory responses.

Consistent with prior studies^5,6^, *Nlrp3* deletion reduced microgliosis in the ARC and both microgliosis and astrogliosis in the DMH (Figure 7). Suppression of microgliosis was RGS10-dependent, being lost in dKO mice in both regions (Figure 7C and E). ARC astrogliosis was elevated with combined deficiency, whereas the reduction in DMH astrogliosis persisted, indicating that RGS10 control of astrocyte responses is region-specific. Furthermore, loss of RGS10 exacerbated metabolic tissue pathology, resulting in increased liver hypertrophy, steatosis, NF-κB activation, and macrophage infiltration, along with adipose tissue dysfunction and inflammation. Notably, the dependence on RGS10 was pathway-specific: the glycemic (Figure 2I), steatosis (Figure 3D), and microglial (Figure 7) benefits of NLRP3 deficiency were abolished or attenuated in dKO mice, whereas hepatic inflammatory and fibrotic responses (Figures 3F, H, and 4) and ARC astrocyte responses (Figure 7C) were instead worsened by combined deficiency. These findings indicate that RGS10 exerts dominant control over inflammatory signaling and myeloid polarization, functioning primarily upstream of the NLRP3 inflammasome. Consistent with its enrichment in microglia and myeloid cells, RGS10 gates NLRP3-dependent benefits within the cell types in which it is expressed, while astrocytes and fibrotic responses are regulated through parallel pathways. Together, our data establish RGS10 as a key immunometabolic regulator restraining HFD-induced inflammation and metabolic dysfunction across tissues and identify it as a potential modifier of inflammasome-targeted therapies.

Our findings indicate that RGS10 inhibits the NLRP3 inflammasome by modulating Ca^2+^ signaling, though the exact mechanism at each phase remains unclear. In microglia, RGS10 inhibits store-operated Ca^2+^ entry by binding to STIM2, reducing proinflammatory gene expression^28^; consistent with this, extracellular calcium is required for increased priming and activation in *Rgs10*^*−/−*^ BMDMs (Figure 1). Prior work^25^, and our data indicate that RGS10 suppresses inflammasome component expression through NF-κB inhibition. Ca^2+^ signaling is known to modulate NF-κB activity^53,54^ and to promote inflammasome assembly via mitochondrial destabilization^30–32^. RGS10 has been reported to increase cAMP levels and PKA activity while suppressing mTORC1 signaling^55–57^, each of which independently inhibits NLRP3^30,58,59^. Together, these observations suggest that RGS10 may regulate NLRP3 inflammasome activity through multiple converging mechanisms, which warrant further definition.

RGS10 expression is dynamically regulated during inflammatory responses. LPS promotes histone deacetylation-mediated repression of *Rgs10* in microglia and macrophages^18,19^, and aging alters RGS10 expression across immune populations^20^. Reduced RGS10 has been reported in cerebrospinal fluid (CSF) and circulating immune cells from individuals with PD^46^. Consistent with this, we found RGS10 declines in aged and PD human microglia, and aged *Rgs10*^*−/−*^ mice spontaneously develop glucose intolerance and increased weight (Figure 8), suggesting that persistent inflammatory and age-related signals remove an important brake on immune activation.

NLRP3 inhibition has advanced clinically, with several small-molecule inhibitors in early development^60,61^. In diet-induced obesity models, the brain-penetrant NLRP3 inhibitor NT-0796 reduced body weight and fat mass while reversing both peripheral and hypothalamic neuroinflammation^5^. Moreover, co-administration of semaglutide enhanced weight loss and more effectively suppressed inflammation than either alone^6^. Our findings suggest that RGS10 may represent a previously unrecognized determinant of therapeutic responsiveness. Because the metabolic protection conferred by NLRP3 loss depended on RGS10, reduced RGS10 expression, as occurs with aging or chronic inflammation, may limit the efficacy of inflammasome-targeted interventions. Future studies should determine whether increasing RGS10 expression can attenuate aging-related or diet-induced chronic systemic inflammation and improve outcomes of pharmacologic NLRP3 inhibition.

## Methods

### Animals

*Nlrp3*^*−/−*^, *Rgs10*^*−/−*^, *Nlrp3*^*−/−*^/*Rgs10*^*−/−*^ (dKO), and WT mice were bred in-house in the C57BL/6 background. Male (n=11–17) and female (n=5–6) mice were fed HFD consisting of 60% kcal from fat (Research Diets D12492i) or NC for 12 weeks. Mice were housed in a temperature-controlled environment with a 12 h light/dark cycle with free access to food and water. Food consumption and weight were recorded every 3–4 days. After 11 weeks, glucose tolerance and insulin sensitivity tests were performed. After 12 weeks, mice were euthanized using isoflurane and cervical dislocation for tissue collection. All research was performed in accordance with the NIH guidelines for Animal Care and Use and approved by the Institutional Animal Care and Use Committee at the University of Georgia.

### Cell culture

Bone marrow was isolated from the femur and tibiae of WT and *Rgs10*^−/−^ mice aged 9–16 weeks as described previously^17^. For each preparation, marrow was pooled from 3 mice of the same genotype, and three independent preparations were generated on separate days. Cells were cultured for 7 days at 37°C with 5 % CO_2_ in Dulbecco’s modified Eagle’s medium nutrient mixture F12 (DMEM/F12) supplemented with 10 % fetal bovine serum (FBS) (R&D), 1 % penicillin/streptomycin (Corning),1 % L-glutamine (Sigma), and 20 % L-929 conditioned medium. BMDMs were plated in 12-well cell culture plates (1.0 × 10^6^ cells/well) without L-929 medium for 2 days to deprive them of macrophage colony-stimulating factor (M-CSF). On the day of the experiment, the culture medium was replaced with 600 μL of fresh medium (CON) or fresh medium containing 100 ng/mL LPS, and cells were incubated for 3 h. Inflammasome activation was induced in serum-free medium by treating cells with 5 mM ATP for 2 h or 5 μM nigericin for 1 h. Cell lysates and supernatants were utilized to measure inflammasome priming and activation.

### Western Blot Analysis

Proteins were precipitated from BMDM supernatants with a 4:1 mixture of methanol and chloroform as previously described^62^. Protein precipitates were resuspended in 50–100 μL 1x Laemmli sample buffer (Bio-Rad) containing 1x protease inhibitor mix (Pierce #032955). The corresponding cells were lysed directly in 100 μL of the same buffer. Samples were heated to 95 °C for 5 min. Fresh liver and adipose tissues were homogenized by sonication in 1 × RIPA buffer (Millipore Sigma #20–188) containing protease inhibitors (Pierce #32955) and phosphatase inhibitors (Pierce #A32957). Homogenates were incubated on ice for 20 min with intermittent vortexing, then centrifuged for 5 min at 10,000 × g at 4 °C. Supernatants were collected, and protein concentrations were quantified using a BCA protein assay kit (Pierce #23227). Lysates were diluted in Laemmli buffer (Bio-Rad) and 1X RIPA buffer and heated at 70 °C for 10 min. Tissue homogenates (20–25 μg of total protein), BMDM lysates, and equal volumes of precipitated BMDM supernatants were separated on 4–20 % pre-cast SDS electrophoresis gels (Bio-Rad) and transferred onto 0.45 μm PVDF membranes (Millipore). Membranes were blocked with 5 % milk in TBST, then probed for the primary antibodies overnight at 4 °C, washed with TBST, and incubated with the appropriate HRP-conjugated secondary antibody (1:2000; Jackson ImmunoResearch Lab). Blots were developed with SuperSignal West Pico or Femto substrates (Thermo Fisher Scientific), imaged on a Bio-Rad ChemiDoc^™^ MP, and quantified in Image Lab (Bio-Rad). BMDM lysates and supernatants were normalized to stain-free total protein. Liver was normalized to β-actin and ingWAT to vinculin. The primary antibodies (1:1000 unless noted): NLRP3 (Adipogen #AG-20B-0014), IL-1β (R&D #AF-401-NA, 1:800), GSDMD (Cell Signaling #39754S), and caspase-1 (Adipogen #AG-20B-0042), IκBa (Cell Signaling #4814T), CD68 (Abcam #ab283654), NF-κB p65 (Cell Signaling #8242T), NF-κB phospho-p65 (Cell Signaling #3033S), p62 (Cell Signaling #39749T), phospho-p62 (Cell Signaling #16177T), β-actin (Santa Cruz Biotechnology #sc-47778), vinculin (Santa Cruz Biotechnology #sc-73614).

### ELISA

IL-1β secreted in BMDM supernatants was measured using the mouse IL-1β ELISA MAX^™^ kit (BioLegend #432615) according to the manufacturer’s protocol. Briefly, supernatants were collected and loaded on 96-well plates (Corning #9018) that were coated with 100 μL capture antibody per well overnight at 4 °C. Plates were washed and blocked with 200 μL 1x diluent for 1h. Plates were washed and incubated with 100 μL standards and samples for 2 h. Plates were washed and incubated with 100 μL detection antibody for 1 h, followed by washing and 100 μL Avidin-HRP for 30 min. Plates were washed and incubated in the dark with 100 μL Substrate Solution D for 15 min. The reaction was stopped with 50 μL of a 1 M H_3_PO_4_ solution. The plates were read at 450 nm. Data are representative of 3 independent experiments.

### Glucose and insulin tolerance tests

Glucose tolerance tests (GTT) and Insulin sensitivity tests (IST) were performed as previously described^21^ during 11 weeks of feeding, separated by 3– 4 days, with mice fasted for 3 h with free access to water. For GTT, mice received 1–2 g/kg glucose intraperitoneally (Figure 2, **S3)** or by oral gavage (Figure 8), dosed based on total body weight. Tail blood glucose was measured with an AmStrip Plus glucometer at 0, 15, 30, 60, 90, 120, and 180 min. For IST, mice received 0.75–1.0 IU/kg insulin (Sigma #I5523) intraperitoneally. Tail blood was measured at 0, 15, 30, 60, 90, 120, and 180 min. IST and GTT tests were performed 3–4 days apart to allow for recovery. Analysis tables for Figure 2 and **Figure S3** are shown in **Table S1** (males) and **Table S2** (females).

### Histological analyses of liver and adipose tissues

Liver and adipose tissues were formalin-fixed, paraffin-embedded, cut into 5 μm sections, and stained with hematoxylin and eosin. Images were acquired on a Zeiss Axio Scope. A1 with 40X for liver and 20X for adipose tissue, with non-overlapping fields per section. Liver images were analyzed using ImageJ with the LabKit pixel-classification plugin (workflow, Figure 3B). A single classifier was trained on a representative image containing ballooned hepatocytes, lipid vacuoles, and unaffected parenchyma, and applied unchanged to all images. After manual removal of false-positive ROIs (objects were excluded if vascular limina, portal structures, or processing artifacts were present), segmented images were thresholded to yield macrovesicular vacuolar area (MVA) and total vacuolar area. Non-macrovesicular vacuolar area (NMVA) was derived by subtracting macrovesicular from total vacuolar area. Both are expressed as a percent of total tissue area. Because neutral lipid is extracted during paraffin processing, these measures quantify optically empty vacuolar space rather than lipid content directly. NMVA is an operational morphometric measure of microvesicular and hydropic changes; it is not equivalent to the categorical hepatocyte ballooning criterion of the NSA/SAF system, which requires cell-level assessment of hepatocyte enlargement and cytoplasmic rarefaction. Adipose sections were analyzed using ImageJ with the Adiposoft plugin (minimum diameter 20 μm, maximum 300 μm) as previously described^63^, using manual-corrected mode with edge-touching cells excluded. Adipocyte number, mean cross-sectional area, and the size-frequency distribution are reported.

### Quantitative real-time PCR

Liver and adipose tissues from WT, *Nlrp3*^−/−^, *Rgs10*^−/−^, and dKO mice fed NC or HFD were minced in cold PURY Cell and Tissue Lysis Buffer, homogenized by sonication, and centrifuged at 20,000 × g for 3 min. RNA was isolated from the supernatant with on-column gDNA removal (PURY RNA Plus, GenDEPOT #P2030–050) per the manufacturer’s protocol. Isolated RNA was pooled (n=4–5 mice per genotype per diet) at equal concentrations by genotype. 700 ng (adipose tissues) or 4 μg (liver) of RNA per sample was reverse transcribed with amfiRivert cDNA Synthesis (GenDEPOT #R5600–100). RT-qPCR was performed in triplicate with SYBR Green in 384-well plates on a QuantiStudi 6 Flex (Thermo Fisher Scientific). The relative mRNA levels were quantified using the 2^−ΔΔCT^ method, normalized to the geometric mean of *Gapdh, 18s*, and *cyclophilin*. RT-qPCR primers (Integrated DNA Technologies, Coralville) are listed in **Table S3**.

### Isolation of stromal vascular fraction and flow cytometry

Fresh ingWAT and eWAT from WT (n=4), *Rgs10*^−/−^ (n=4), *Nlrp3*^−/−^ (n=4), and dKO (n=3) mice were collected. For each animal, ingWAT and eWAT were combined, minced, and digested in 10 mL sterile-filtered DMEM with 2 % bovine serum albumin (BSA) (Fisher) and 1 mg/ml collagenase type I (Gibco) for 15–30 min at 37 °C with agitation for tissue digestion. Digestion was stopped with 1 mL FBS, and samples were centrifuged at 440 × g for 5 minutes to remove floating adipocytes from the stromal vascular fraction (SVF). The SVF cell pellet was resuspended and filtered through 70 μm cell strainers, washed with 10 mL FACS buffer, and pelleted again. Cells were stained for 20 min at 4 °C in BD Horizon Brilliant Stain Buffer (#563794) with TruStain FcX^™^ anti-mouse CD16/32 antibody (BioLegend, #101320, 1:100) to block Fc receptors, and fluorophore-conjugated antibodies (all 1:200) against CD45 (BioLegend, #103126), Ly6G (eBioscience, #56-5931-82), CD11b (BioLegend, #101208), CD115 (BioLegend, #135510), CD206 (BioLegend, #141703), CD11c (BioLegend, #117336), and Ly6C (BioLegend, #128030). After staining, cells were washed twice with FACS buffer and analyzed using a NovoCyte Quanteon Flow Cytometer (Agilent Technologies) with NovoExpress flow cytometry software. Compensation was performed with single-stained beads, and gates were set using FMO controls.

### Immunofluorescence analysis

Perfused brains were post-fixed in 4 % PFA for 24 h, cryopreserved in 30 % sucrose solution. Brains were sectioned coronally at 30 μm on a cryostat; sections collected in 1 in 8 series. Free-floating brain sections underwent antigen retrieval overnight at 60°C in citrate buffer (0.1 M sodium citrate and 0.1 M citric acid), were washed in PBS and quenched in 0.2 M glycine for 1 h, permeabilized for 35 minutes in TBS with 3 % TritonX-100 containing 1 % normal serum and blocked for 1 h at room temperature in TBS with 1% normal serum. Sections were incubated overnight at 4 °C with Iba1 (Wako, #019–19741, 1:500) and GFAP (Synaptic Systems, #173 308, 1:1000) in TBS with 1 % TritonX-100 and 1 % normal serum, washed in TBST (TBS with 0.05 % TritonX-100) and incubated for 2 h at room temperature with Alexa FluorÒ secondary antibodies in TBS with 1 % TritonX-100 and 1 % normal serum. Nuclei were labeled with Hoescht33258 (Invitrogen #H3569, 1:10,000) for 5 minutes, and sections were mounted in ProLongÔ Gold antifade (Invitrogen #P3690). Images were acquired on a Keyence BZ-X800 with identical exposure and gain settings across all animals. Up to 4 images per animal were taken with a field covering the dorsomedial hypothalamus (DMH) and ventromedial hypothalamus (VMH) (20× objective) and the arcuate nucleus (ARC) (40× objective). Images were analyzed using ImageJ software using a fixed threshold applied identically to all images within a channel to measure total immunoreactive area per identical ROI. Total immunoreactive area of each ROI for all images was plotted for analysis.

### Statistical methods

Data were analyzed using GraphPad Prism 10 (GraphPad Software, Inc.; San Diego, CA, United States). Data are presented as mean ± SEM. Comparisons between treatment groups were performed using two-way ANOVA followed by Tukey’s multiple comparisons test or Fisher’s LSD post-hoc test. Comparisons within groups were analyzed using one-way ANOVA or Student’s unpaired t-test. A p-value of < 0.05 was considered statistically significant. Correlation Analyses were performed using GraphPad Prism 10 software.

## Supplementary Material

Supplementary Files

This is a list of supplementary files associated with this preprint. Click to download.

- GreshametalSupplementarydata.pdf

## Figures and Tables

**Figure 1.**
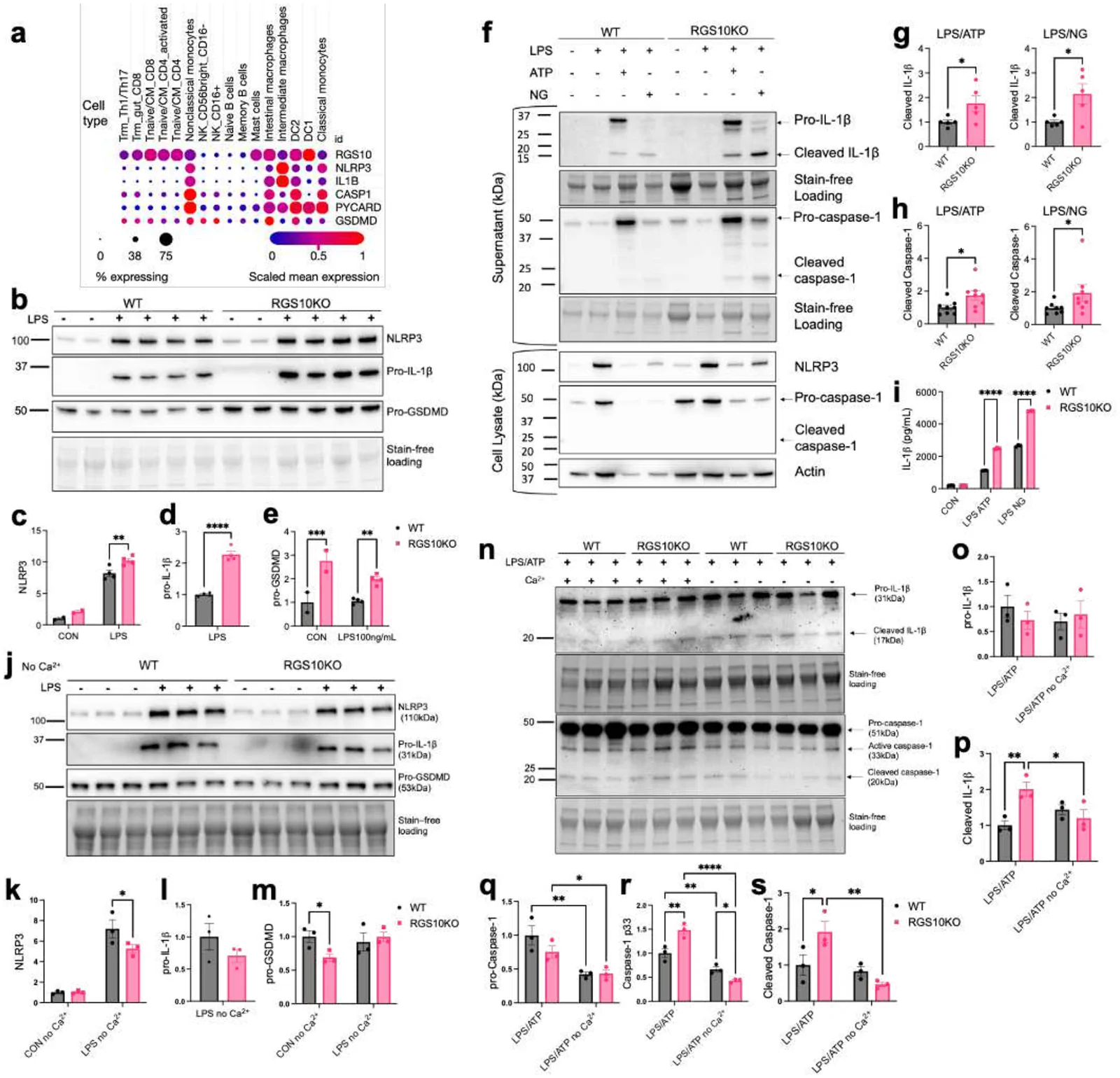
RGS10 deficiency enhances NLRP3 inflammasome priming and activation in BMDMs in a calcium-dependent manner. BMDMs were generated from male WT and *Rgs10*^*−/−*^ mice; bone marrow was pooled from 3 mice per preparation, and n=3 independent preparations per genotype unless noted. **(A)** Expression of *RGS10*, *NLRP3*, *IL1B*, *CASP1*, *PYCARD* (ASC), and *GSDMD* in human immune cells, from the Broad Institute Single Cell Portal (accessed May 14, 2026^23,24^). **(B, J)** Representative Immunoblots of NLRP3, pro-IL-1β, and pro-GSDMD in lysates of BMDMs treated with LPS (100 ng/mL, 3 h) in complete medium **(B)** or calcium-free medium **(J)**. **(C-E, K-M)** Quantification of NLRP3 **(C, K)**, pro-IL-1β **(D, L)**, and pro-GSDMD **(E, M)**, normalized to stain-free total protein and expressed relative to WT CON/WT LPS. **(F)** Representative immunoblots of lysates and supernatants from BMDMs primed with LPS (100 ng/mL, 3 h) and stimulated with ATP (5mM, 2 h) or nigericin (NG, 5uM, 1 h). **(G, H)** Quantification of secreted IL-1β (G) and caspase-1 p20 **(H)** released into the conditioned medium normalized to stain-free total protein and expressed relative to WT. **(I)** Secreted IL-1β ELISA in conditioned medium following LPS/ATP or LPS/NG stimulation. **(N)** Representative immunoblots of supernatants from LPS-primed BMDMs stimulated with ATP in the presence or absence of extracellular calcium. **(O-S)** Quantification of pro-IL-1β **(O)**, cleaved IL-1β p17 **(P)**, pro-caspase-1 **(Q)**, caspase-1 p33 **(R)**, and caspase-1 p20 **(S)**. Lysate blots were normalized to stain-free total protein. Statistical analyses were performed using two-way ANOVA with Fisher’s LSD **(C, E, I, K, M, O, P, Q, R, S)** or unpaired *t*-test **(D, G, H, L)**. Data are shown as mean ± SEM. *p < 0.05, **p < 0.01, ***p < 0.001, ****p < 0.0001.

**Figure 2.**
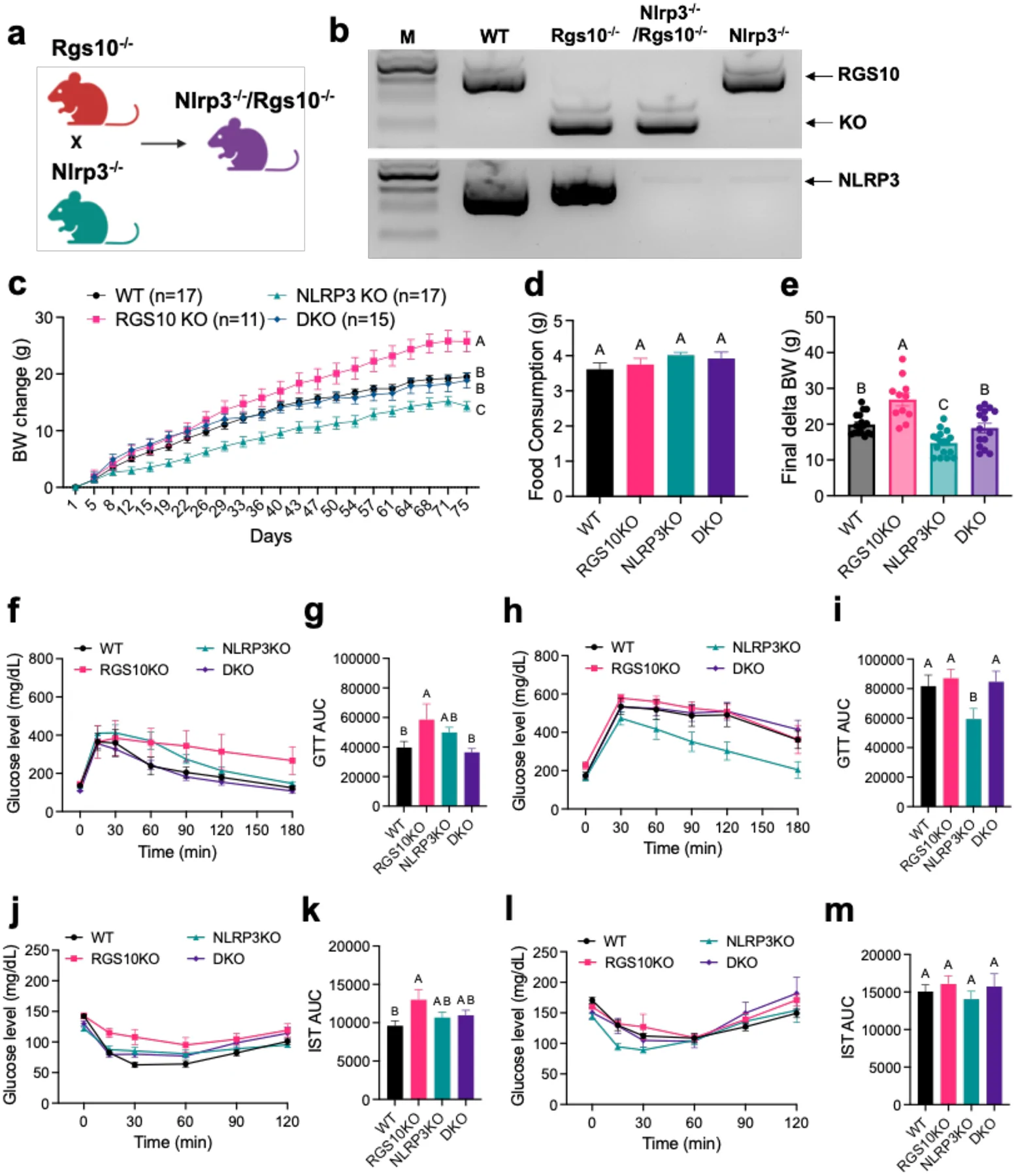
NLRP3 deficiency protects against HFD-fed weight gain and glucose intolerance in an RGS10-dependent manner in male mice. **(A)** Breeding scheme used to generate *Nlrp3*^*−/−*^/*Rgs10*^−/−^ (dKO) mice from *Nlrp3*^−/−^ and *Rgs10*^−/−^ lines. **(B)** Representative genotyping PCR for WT, *Rgs10*^−/−^, *Nlrp3*^−/−^, and dKO genotypes. **(C-E)** Male mice of each genotype were fed HFD for 12 weeks (n=11–17): cumulative body weight changes **(C)**, average daily food intake per mouse **(D)**, and final body weight **(E). (F-M)** Glucose tolerance (GTTs; **F-I**) and insulin sensitivity tests (IST, **J-M**) were performed during week 11 after a 3 h fast, in mice fed NC (n=5–8) **(F, G, J, K)** or HFD **(H, I, L, M)**. **(G, I, K, M)** area under the curve. Statistical analyses were performed by two-way ANOVA (main column effect) with Tukey’s post-hoc test **(C**), Kruskal-Wallis with Dunn's multiple comparisons test **(D)**, one-way ANOVA with Tukey’s post-hoc test **(E)**, mixed effects model with Tukey’s post hoc test **(F, H, J, L)**, or one-way ANOVA with Fisher’s LSD **(G, I, K, M)**. Error bars represent SEM.

**Figure 3.**
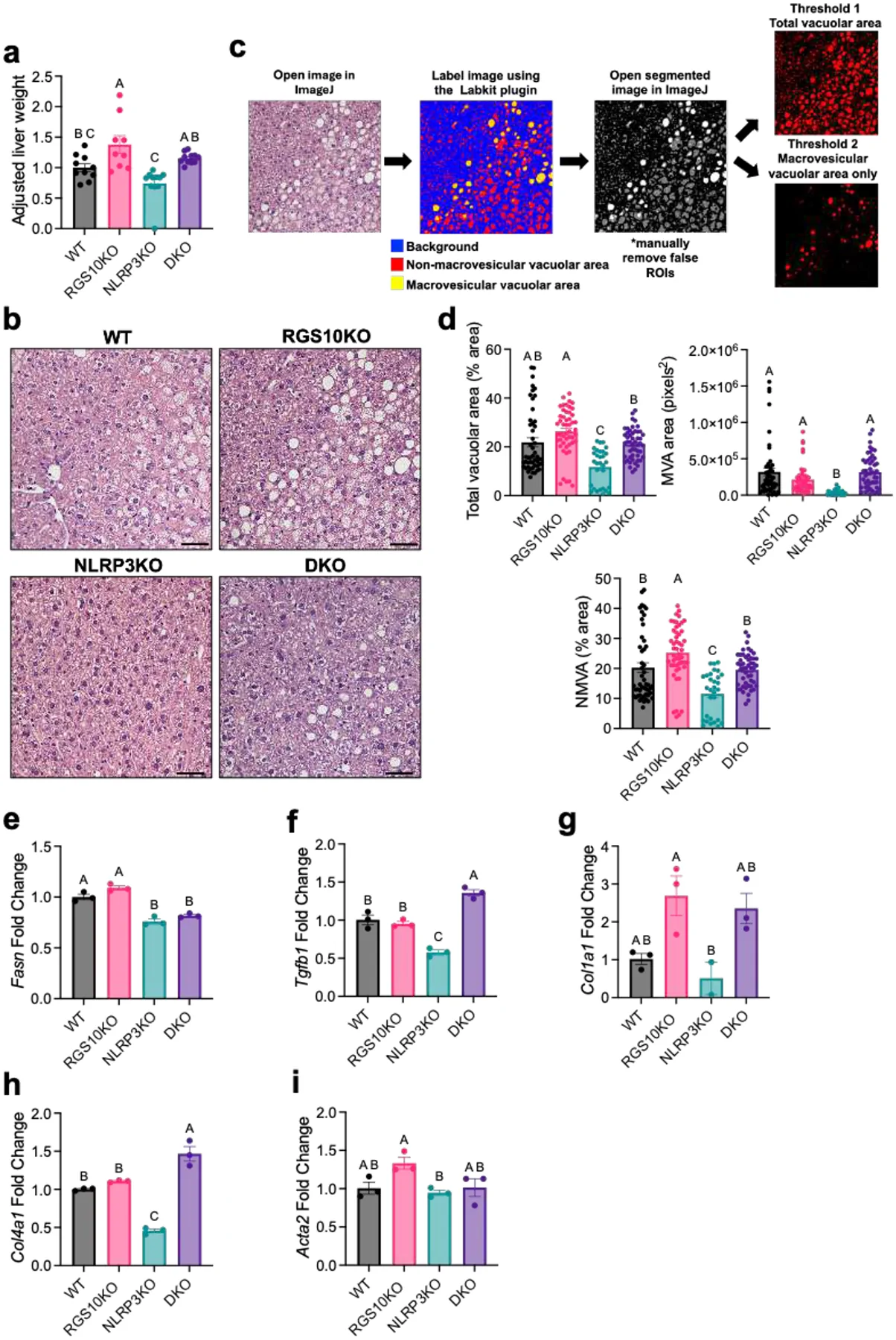
NLRP3 deficiency reduced HFD-induced hepatic steatosis in an RGS10-dependent manner. Livers were collected from WT, *Rgs10*^−/−^, *Nlrp3*^−/−^, and dKO mice after 12 weeks of HFD. **(A)** Liver weights of male mice (n=9–11). **(B)** Representative H&E-stained liver sections (40X objective). Scale bar, 50 μm. **(C)** Image analysis workflow (ImageJ with the Labkit). Sections were segmented by pixel classification into macrovesicular and non-macrovesicular vacuolar compartments: after blinded removal of false-positive ROIs, segmented images were thresholded to yield total vacuolar area and macrovesicular vacuolar area (MVA), and non-macrovesicular vacuolar area (NMVA) was calculated by subtraction. **(D)** Quantification of total vacuolar area, MVA, and NMVA, expressed as percent of tissue area. Ten fields were analyzed per animal (WT (n=5), *Rgs10*^−/−^ (n=5), *Nlrp3*^−/−^ (n=3), and dKO (n=5). **(E-I)** Expression of *Fasn* **(E)**, *Tgfb1* **(F)**, *Col1a1* **(G)**, *Col4a1* **(H)**, and *Acta2* **(I)** transcript levels by RT-qPCR (n=5 mice per genotype). Data are mean ± SEM, analyzed using one-way ANOVA with Tukey’s post-hoc test.

**Figure 4.**
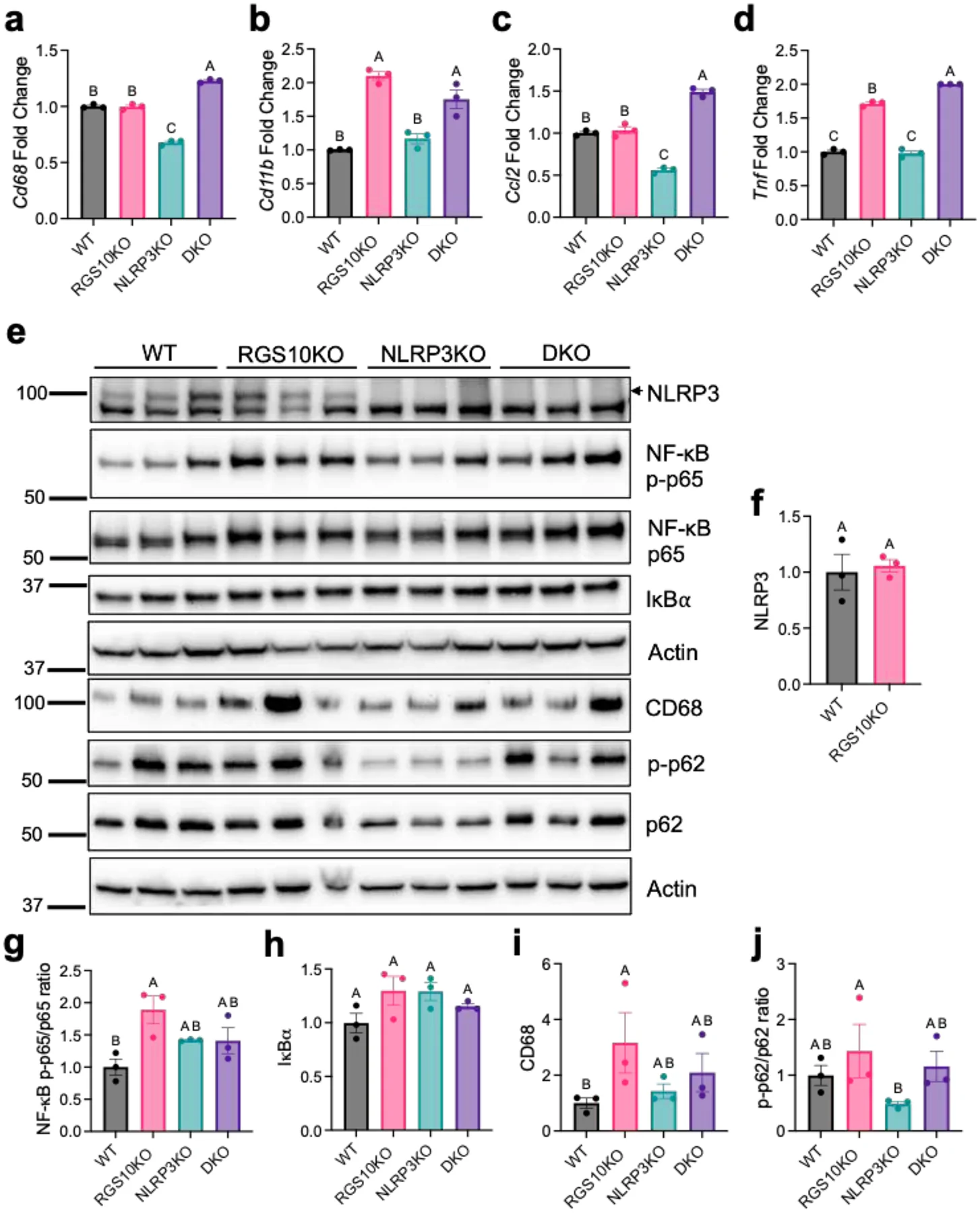
RGS10 deficiency exacerbates HFD-induced hepatic inflammation and NF-κB activation, and combined RGS10 and NLRP3 deficiency does not restore it. Livers were collected from male WT, *Rgs10*^−/−^, *Nlrp3*^−/−^, and dKO mice after 12 weeks of HFD. **(A-D)** Hepatic *Cd68* **(A)**, *Cd11b* **(B)**, *Ccl2* **(C)**, and *Tnf* **(D)** transcripts by RT-qPCR (pooled n=5 per genotype, ran in triplicate), normalized to the geometric mean of three housekeeping genes and expressed relative to WT **(E)** Representative immunoblots of hepatic of NLRP3, NF-κB phospho-p65/p65 ratio, IκBa, CD68, and phospho-p62 and p62. **(F-J)** Quantification of the relative protein levels of NLRP3 **(F)**, phospho-p65/p65 **(G)**, IκBa **(H)**, CD68 **(I)**, and phospho-p62/p62 **(J)**, normalized to b-actin and expressed relative to WT (n=3 mice per genotype). Data are mean ± SEM, analyzed using one-way ANOVA with Tukey’s post hoc test or Fisher’s LSD.

**Figure 5.**
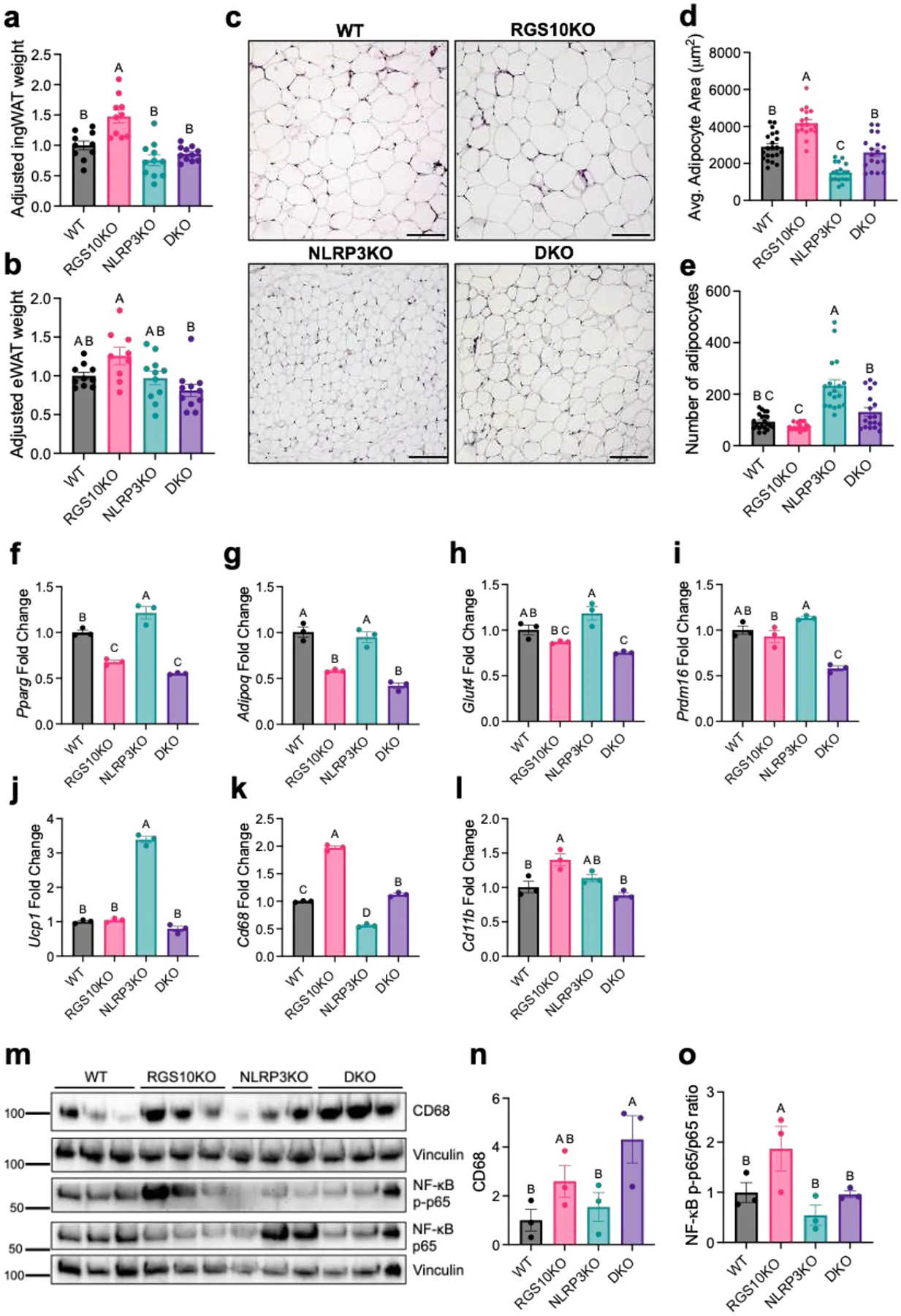
RGS10 is required for NLRP3-dependent adipose remodeling but independently restrains adipose inflammatory gene during HFD. Inguinal (ingWAT) and epidydimal (eWAT) white adipose tissues were collected from WT, *Rgs10*^−/−^, *Nlrp3*^−/−^, and dKO mice after 12 weeks of HFD. **(A-B)** ingWAT **(A)** and eWAT **(B)** mass (n=10–11 mice per genotype). **(C)** Representative of H&E-stained ingWAT. (20x objective; n=3 per genotype). Scale bar, 150μm. **(D, E)** Mean adipocyte cross-sectional area **(D)** and number of adipocytes **(E)** in ingWAT, quantified with Adiposoft (n=5–7 images per animal). **(F-L)** Expression of *Pparg* **(F),** *Adipoq* **(G)**, *Glut4* **(H)**, *Prdm16* **(I**), *Ucp1* **(J)**, *Cd68* **(K)**, and *Cd11b* **(L)** as measured by RT-qPCR (n=5). **(L-N)** Western blot analysis **(G)** and quantification of the relative protein levels of CD68 **(H)** and NF-κB phospho-p65/p65 ratio **(I)** (n=3), normalized to vinculin and expressed relative to WT. Data are mean ± SEM, analyzed using one-way ANOVA with Tukey’s post-hoc test or Fisher’s LSD.

**Figure 6.**
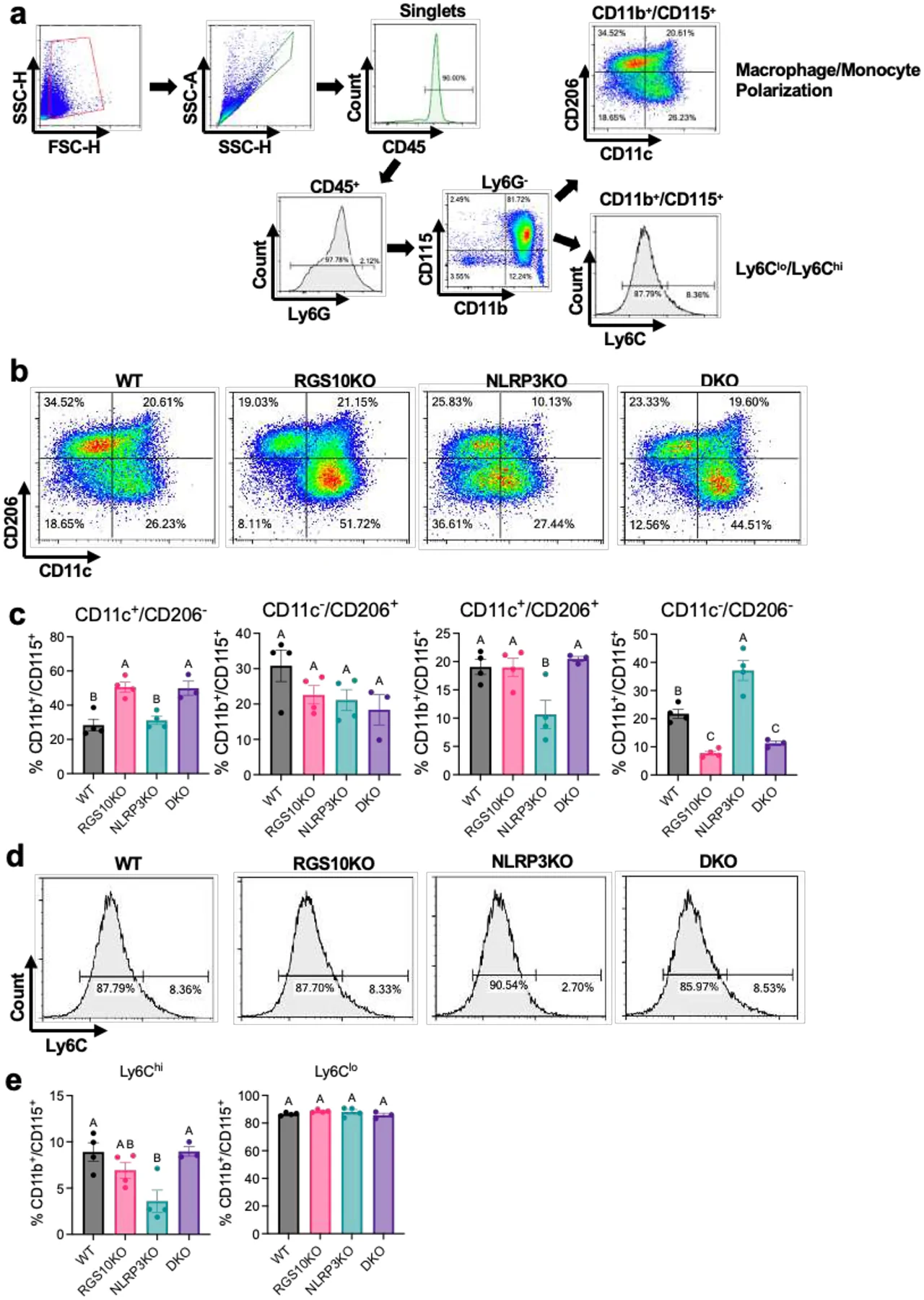
RGS10 shapes adipose myeloid composition independently of NLRP3 and is required for the anti-inflammatory monocyte effect of NLPR3 deficiency. Combined stromal vascular fractions were isolated from ingWAT and eWAT tissues of from WT (n=4), *Rgs10*^−/−^ (n=4), *Nlrp3*^−/−^ (n=4), and dKO (n=3) mice after 12 weeks of HFD. **(A)** Gating strategy to analyze stromal vascular fraction of adipose tissue. **(B)** Representative density plots of CD11c and CD206 from CD11b^+^/CD115^+^ populations. **(C)** Frequencies of monocyte/macrophage subpopulations. **(D)** Representative histograms of Ly6C expression from CD11b^+^/CD115^+^ populations. **(E)** Frequencies of Ly6C subpopulations. Data are mean ± SEM, analyzed using one-way ANOVA with Tukey’s post-hoc test.

**Figure 7.**
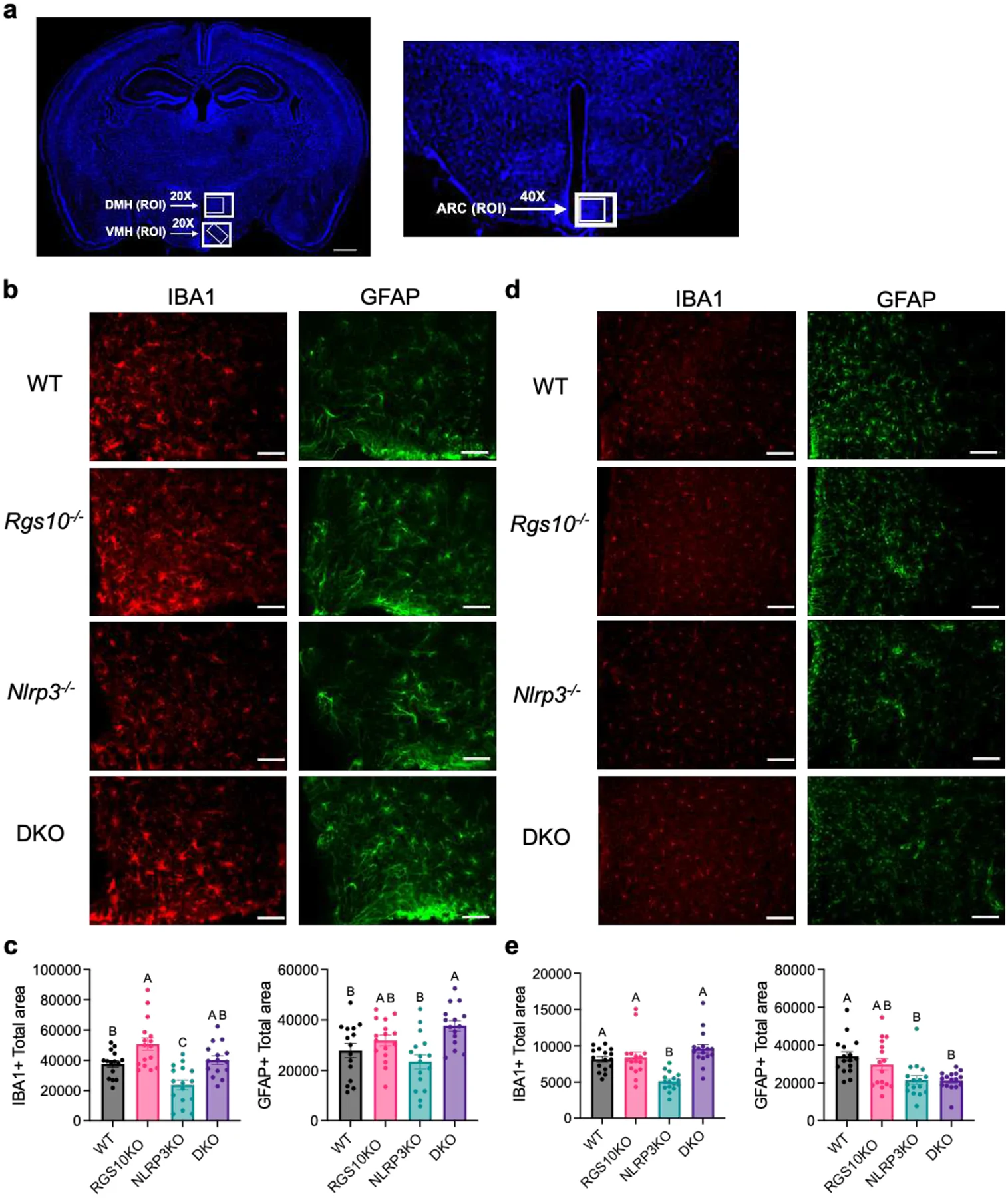
RGS10 is required for the suppression of hypothalamic gliosis conferred by NLRP3 deficiency in HFD-fed mice. **(A)** Representative brain section showing regional analysis of the ARC, DMH, and VMH regions, delineated using the mouse brain atlas. Scale bar: 500 μm. **(B, D)** Representative immunofluorescence images of Iba1 (microglia) and GFAP (astrocytes) in the ARC (**B**; 40X objective, scale bar: 50 μm) and DMH **(D;** 20X objective, scale bar: 100 μm) of male WT, *Rgs10*^*−/−*^, *Nlrp3*^*−/−*^, and DKO mice after 12 weeks of HFD (n=4 mice per genotype). **(C, E)** Iba1+ area or GFAP+ area per ROI in the ARC **(C)** and DMH **(E)** from all images (n=3–4 images per animal). Data are mean ± SEM, analyzed using one-way ANOVA with Tukey’s post-hoc test.

**Figure 8.**
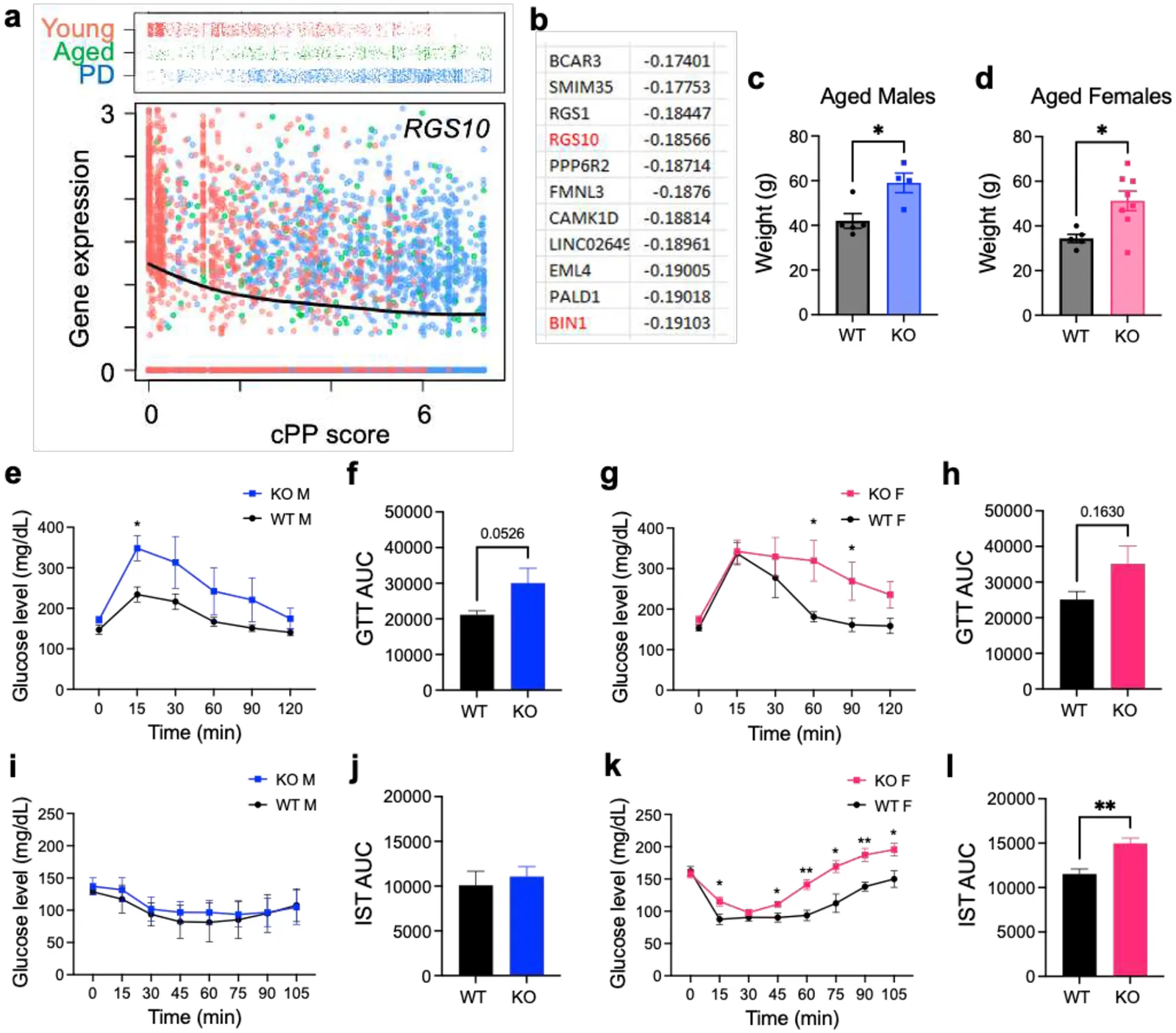
RGS10 declines in human microglia with aging and PD, and its loss drives sex-dimorphic age-related metabolic dysfunction. **(A)** Reanalysis of published human microglial snRNA-seq data^44^ showing *RGS10* expression across the continuous pseudopathology (cPP) trajectory, which captures the transition from homeostatic microglia to aging- and PD-associated inflammatory states. Points represent individual cells colored by group (Young, Aged, and PD). **(B)** *RGS10* ranked among genes negatively associated with cPP trajectory, shown with neurodegeneration-associated genes including *BIN1*. **(C, D)** Body weight of aged (17–21 months) male (n=4–5 per group) **(C)** and female (n=5–8 per group) **(D)** WT and *Rgs10*^*−/−*^ mice. **(E-L)** GTT **(E-H)** and IST **(I-L)** tests of aged (17–21 months) male (n=4–5 per group) **(E, F, I, J)** and female (n=5–8 per group) **(G, H, K, L)** WT and *Rgs10*^*−/−*^ mice. Statistical analyses were performed by unpaired t-test **(C, D, F, H, J, L)** or two-way ANOVA with Fisher’s LSD **(E, G, I, K)**. Data represent mean ± SEM. *p < 0.05, **p < 0.01.

## Data Availability

The data supporting the findings of this study are available from the corresponding authors upon request.
